# An Independent Evaluation of the Psychometric Properties of the Russian Version of the Pediatric Daytime Sleepiness Scale (PDSS)

**DOI:** 10.11621/pir.2023.0314

**Published:** 2023-09-30

**Authors:** Ilya M. Zakharov, Victoria I. Ismatullina, Pavel V. Kolyasnikov, Julia A. Marakshina, Artem S. Malykh, Anna O. Tabueva, Timofey V. Adamovich, Marina M. Lobaskova, Sergey B. Malykh

**Affiliations:** a Ural Federal University named after the first President of Russia B.N. Yeltsin, Yekaterinburg, Russia; b Psychological Institute of Russian Academy of Education, Moscow, Russia; c Lomonosov Moscow State University, Russia

**Keywords:** daytime sleepiness, adolescents, sleep-related problems, sleep duration, psychometric analysis

## Abstract

**Background:**

The quality of sleep significantly impacts children’s day-to-day performance, with at least 20% reporting issues with sleepiness. Valid tools for assessing the quality of sleep are needed.

**Objective:**

In this study, we assessed the psychometric properties of the Russian version of the Pediatric Daytime Sleepiness Scale (PDSS). The initial adaptation of the PDSS was conducted on a sample from the Arctic regions of Russia. This location may have influenced the scale’s generalizability due to variations in natural daylight across different areas of the country.

**Design:**

To rectify this, we gathered a comprehensive, geographically diverse sample from Russia. This combined dataset comprised 3772 participants between 10 to 18 years of age, from nine different regions of Russia.

**Results:**

We confirmed the unifactorial structure of the PDSS, which showed no regional effects. The psychometric analysis indicated that one item from the 8item PDSS could be removed, thereby improving the scale’s model fit. We also observed gender and age impacts on sleep quality: boys reported fewer sleep-related issues than girls, and younger children reported fewer problems than older children.

**Conclusion:**

This study validates the usefulness and reliability of the Russian version of the PDSS, thereby enhancing its general applicability. Furthermore, we replicated previously reported age and sex effects on the sleep quality of school-aged children.

## Introduction

Numerous factors contribute to individual differences in educational achievements, including cognitive attributes, socioeconomic status, and even physical activity levels. However, a frequently overlooked factor is problems with sleep quality, such as sleep deprivation or excessive daytime sleepiness. Prior studies have linked these issues to significant impairments in cognitive performance and learning abilities (Dimitrova & Genov, 2020; Dijk et al., 2012; Liu et al., 2012; Fakier & Wild, 2011). These effects are more pronounced in children and adolescents compared to adults. For instance, in the United States, a minimum of 20% of adolescents report daily sleepiness problems, such as falling asleep at school or while doing homework, excessive tiredness, or issues resulting from oversleeping (National Sleep Foundation, 2006). Student sleepiness has been negatively correlated with academic outcomes in multiple studies (Turner et al., 2019; Jalali et al., 2020; Gallego-Gomez, 2021).

Alongside its cognitive effects, sleep deprivation heightens feelings of anger toward oneself and others (Kamphius et al., 2012; Krizan & Hisler, 2019) and leads to more frequent illness (Orzech et al., 2013). Moreover, excessive daytime sleepiness is often exhibited by children and adolescents (Dorofaeff & Denny, 2006). Significant age effects are also observed within adolescent groups: studies show a twofold decrease (from 50% to 24%) in getting sufficient sleep time when comparing 8th graders to 6th graders (Volmer et al., 2012; Porcheret et al., 2018). This increase in sleepiness is associated with behavioral factors, such as the “eveningness” chronotype found in adolescents residing in brightly lit urban areas (Vollmer et al., 2012). These factors, combined with early school start times, result in a significant sleep deficit with potentially serious implications for student learning quality (Porcheret et al., 2018).

A multi-site study involving 9,000 students from eight public high schools in a three-year project underscored the significant impact of improved sleep (Wahlstrom et al., 2014). The study found that when high schools began at 8:30 AM or later (as compared to the average start time of between 7:45 AM and 8:15 AM), more than 60% of students managed to get at least eight hours of sleep per school night. This led to enhancements in both their health status and academic performance. Teens reporting less than eight hours of sleep had a significantly higher risk of poor decision-making relating to substance use and displayed more symptoms of depression. Conversely, school attendance rates, grades in core subjects such as math, English, science, and social studies, and performance on state and national achievement tests improved with later school day start times. Interestingly, the shift in school start times also had indirect effects, such as a 70% reduction in car crashes for teen drivers 16 to 18 years old.

Given the significance of sleep quality for school children, a reliable tool for measuring it is essential. Timely screening for sleep problems can mitigate severe repercussions and assist in identifying at-risk children. Prior studies have indicated that quick, inexpensive, and robust screening is feasible using subjective measures of sleepiness. Among the available self-rating sleep scales, the Pediatric Daytime Sleepiness Scale (PDSS) has demonstrated the highest validity and reliability (Canto et al., 2014). The PDSS, designed for children ages 11 to 15, comprises eight sleep-related behavior questions and is used to assess sleep-related problems and confirm sleeping habits (Pereira et al., 2010). Successful adaptations of the PDSS exist in German, Brazilian, Turkish, Chinese, Korean, and Japanese versions (Schneider & Randler, 2009; Felden et al., 2016; Bektas et al., 2016; Yang et al., 2010; Rhie et al., 2011; Komada et al., 2016).

The PDSS was previously translated into Russian by Randler and colleagues (Randler et al., 2019) and exhibited satisfactory factor structure, as supported by confirmatory factor analysis (CFA) and reliability (intraclass coefficient = 0.70). However, a unique aspect of that study was that it was conducted in Russian Arctic cities, specifically the Karelia and Murmansk regions, where daylight varies drastically throughout the year. For instance, in Murmansk, the sun remains continuously above the horizon (a phenomenon known as the midnight sun) for over two months. This significant variation in natural light has been shown to influence both mood and sleep patterns, although the results have been inconsistent (see Overland et al., 2020). Notably, most studies examining the effect of daylight variation have focused on adults. Given the results of the “Minnesota sleep study,” it is highly probable that daylight variation across different geographical regions may significantly impact schoolchildren’s academic performance. Collectively, the special characteristics of these studies underscore the need to directly compare sleep quality and its association with academic performance among adolescents.

The objective of the current study was to explore the factors that may affect the sleep quality of Russian schoolchildren. To achieve this, we planned to 1) assess the psychometric properties of the PDSS using a large-scale, geographically diverse Russian sample; 2) analyze potential regional differences in PDSS scores; and 3) examine potential age and gender differences in the sleep quality of Russian schoolchildren. Initially, the PDSS questionnaire was designed for early adolescents 1015 years old. The additional objective of our study was to test whether this age range could be extended up to 18 years old.

## Methods

### Participants

Our study was carried out during 20202021. The combined dataset included 3772 participants; after deleting incomplete data, 3583 participants remained. The gender identity was based on self-reports with the options “Male,” “Female,” and “Don’t want to answer this question.” In the whole sample, 1661 participants identified themselves as boys and 1912 as girls, with 10 participants refusing to identify their gender. The participants’ ages ranged from 10 to 18 years.

Schools from 10 different geographical Russian regions participated in the study. In each school, there were two different days when the data was collected with the difference between days less than a week. The region with the highest daylight variation was Murmansk (68° 58° 45.01” N; its daylight variation includes more than two months of polar day and one month of the polar night). The region with the least daylight variation was Rostov-on-Don (47°13° 29.50° N), which has daylight variation of 8 to 15.5 hours from winter to summer). The number of participants from each region is presented in *Table 1.*

**Table 1:** The distribution of the participants among Russian regions

Region	Collection dates	N
Khabarovsk region	24 November, 2020 2 December, 2020	559
Krasnoyarsk region	24 November, 2020 3 December, 2020	326
Murmansk region	24 November, 2020 2 December, 2020	145
Novosibirsk region	24, 26 November, 2020 7, 9 December, 2020	424
Omsk region	30 November, 2020 10 December, 2020	260
Perm region	24 November, 2020 2 December, 2020	86
Rostov region	24 November, 2020 9, 10 December, 2020	767
Tyumen region	16 February, 2021	549
Yaroslavl region	24 November, 2020 2 December, 2020	229

The data were collected during the COVID-2019 pandemic, which may have affected the study results. In two out of the nine regions, the COVID-related limitations included schools and other educational institutions directly. Preliminary analysis showed that the inclusion of these two regions did not affect the study results, so the present analysis was performed on the full sample.

The study received approval from the Ethics Committee of the Psychological Institute of the Russian Academy of Education (approval №2020/41). The participants were minors and so informed consent was obtained for each participant from parents or legal guardians.

### Procedure

The questionnaire was presented digitally from either the students’ personal computers or in school computer classes.

#### PDSS Questionnaire.

The PDSS is an 8item questionnaire that was developed to assess extreme daytime sleepiness in schoolchildren. It was previously translated into Russian and tested for comprehension by Randler and colleagues (2019). The interviewee rates the frequency of the behaviors itemized below on a 5point Likert scale, which ranges from 0 (never) to 4 (always). Rankings on all the items are totaled to obtain a total score, which could amount to anything from 0 to 32. A higher score indicates greater day-time sleepiness. The start time of filling in the questionnaire was also added as a separate measure.

The original and translated items are presented in *Table 2.*

**Table 2:** Original and translated versions of the PDSS

**№**	**Item in English**	**Item in Russian**
1	How often do you fall asleep or get drowsy during class periods?	Как часто вы засыпаете или чувствуете сонливость во время занятий?
2	How often do you get sleepy or drowsy while doing your homework?	Как часто вы чувствуете сонливость или сонливость во время выполнения домашнего задания?
3	Are you usually alert most of the day?	Вы обычно бдительны большую часть дня?
4	How often are you ever tired and grumpy during the day?	Как часто вы бываете уставшим и раздражительным в течение дня?
5	How often do you have trouble getting out of bed in the morning?	Как часто вам трудно встать с постели по утрам?
6	How often do you fall back to sleep after being awakened in the morning?	Как часто вы снова засыпаете после пробуждения утром?
7	How often do you need someone to awaken you in the morning?	Как часто вам нужно, чтобы кто-то разбудил вас утром?
8	How often do you think that you need more sleep?	Как часто вы думаете, что вам нужно больше спать?

### Statistical approach

Construct validity was examined through CFA with the following adjustment indicators used as criteria: comparative fit index (CFI), root mean square error of approximation (RMSEA), and standardized root mean square residual (SRMR) (Brown, 2014). We also analyzed the psychometric properties of the PDSS according to the IRT approach. Cronbach’s α and McDonald’s ω (McDonald, 1999) were calculated to estimate the reliability of the PDSS. Regression and correlation analyses were used to investigate the relationship of the PDSS scores with other variables. All analyses were performed in the R language for statistical programming with the packages ‘mirt’, ‘lavaan’, and ‘psych’.

## Results

### Psychometric properties of the Russian version of the PDSS

**Figure 1.**
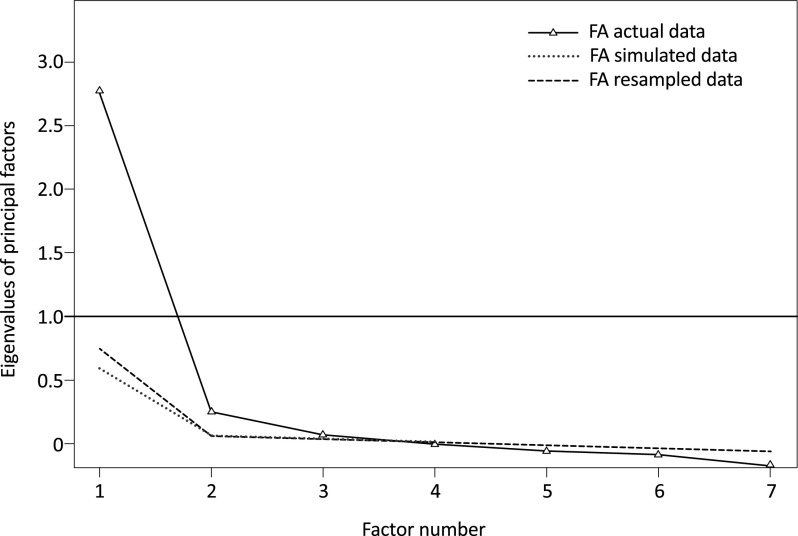
The results of the exploratory factor analysis

*Note. Both scree plot and the fact that there is only one eigenvalue higher than 1 point to a one-factor solution.*

The first step of the analysis was to analyze the factor structure of the questionnaire. To do that, we randomly split our sample into two parts. We first performed an exploratory factor analysis (EFA) on the first part of the sample and then a confirmatory factor analysis (CFA) on the second part. The EFA results pointed to the one-factor solution (*Figure 1*).

The next step of the analysis was to show that the one-factor solution had an adequate fit on the second part of the data. To do that, we fitted a graded response model (the recommended model for ordered polytomous response data) on the whole dataset, using a full-information maximum likelihood fitting function. In addition, we assessed model fit using an index, M2, specifically designed to assess the fit of item response models for ordinal data. We used the M2based root mean square approximation (RMSEA) error as the primary fit index. We also used the standardized root mean square residual (SRMSR) and comparative fit index (CFI) to assess the adequacy of model fit.

The RMSEA value we obtained (RMSEA = 0.09; 95% CI [0.10; 0.12]) was higher than the cutoff value (RMSEA <= 0.06), and the SRMSR value (SRMSR = 0.069) was within the normative value (SRMSR <= 0.08). The CFI (CFI = 0.949) was below the recommended 0.95 threshold. To improve the quality of the model, we used the IRT model to analyze each item’s parameters separately. The results of the IRT analysis are presented in *Table 3.*

**Table 3:** The slope values (a-parameter) and the location parameters (b-parameters) of the items according to the IRT analysis

Item	a	b1	b2	b3	b4
1. Fall asleep/drowsy during class	2.40	–1.65	–0.51	0.55	1.69
2. Drowsy/asleep during homework	1.82	–1.23	–0.13	0.90	1.96
3. Usually alert during the day (reverse coded)	1.32	–1.07	0.80	1.93	3.60
4. Tired and grumpy during the day	1.79	–2.24	–0.39	0.82	2.13
5. Trouble getting out of bed in the morning	1.56	–1.93	–0.75	0.21	1.18
6. Fall back to sleep after being awakened	1.18	–1.48	0.01	1.00	2.31
7. Need someone to awaken you	0.80	–0.79	0.57	1.72	3.00
8. How often do you think you need more sleep?	1.96	–1.48	–0.53	0.23	0.98

The values of the slope (a-parameters) ranged from 0.80 to 2.40. A slope parameter measures how well an item differentiates respondents with different levels of a latent trait. Item 1 was the most discriminating item, and Item 7 was the least discriminating. This result can also be visualized with the category characteristic plot (*Figure 1*). Three location parameters (b-parameters) are also listed for each item. Location parameters are interpreted as the value of theta corresponding to a .5 probability of responding at or above that location on an item. There are m-1 location parameters, where m refers to the number of response categories on the response scale. The location parameters indicate that the responses covered a wide range of latent traits.

**Figure 2.**
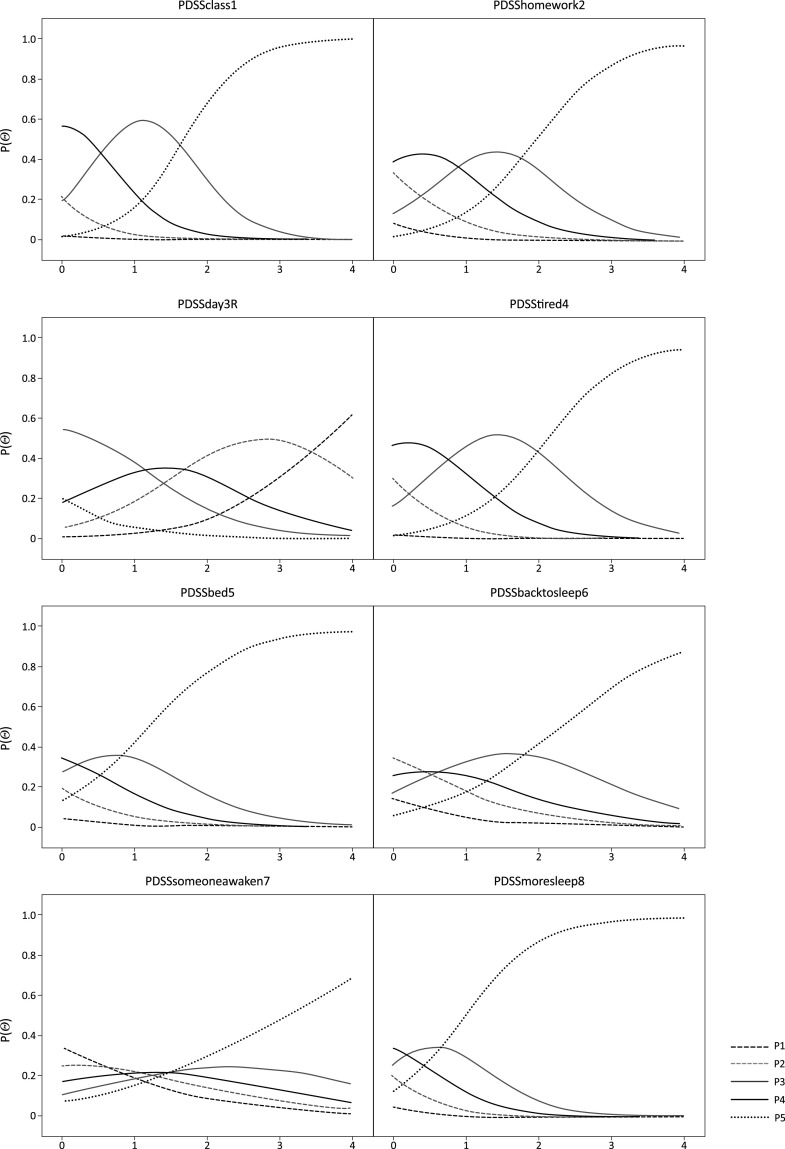
The category characteristic plot for the IRT parameters

*Note. P1P5 = response options (5point Likert scale, P1 = “Never”, P5 = “Always”). P(β) shows the probability of choosing each type of the answer to the questionnaire item.*

Altogether, the IRT analysis shows that Item 7 (“How often do you need someone to awaken you in the morning?”) does not add to identifying individual differences in sleep quality and can be omitted. The model fit improves after deleting this item, with RMSEA equaling 0.09 (95% CI[0.09; 0.10]), SRMSR equaling 0.05, and CFI = 0.96. The difference between the distributions of the original and recalculated (corrected) PDSS total scores are presented in *Figure 2.*

The comparison of the factor analysis and reliability measures (Cronbach’s α and McDonald’s ω) of the original and edited versions of the scale is presented in *Table 4.*

**Table 4:** The factor analysis and reliability measures of the original and edited PDSS

Item	F1 original	h2 original	F1 edited	h2 edited
Fall asleep/drowsy during class	0.816	0.666	0.829	0.687
Drowsy/asleep during homework	0.73	0.533	0.738	0.544
Usually alert during the day (reverse coded)	0.613	0.376	0.62	0.385
Tired and grumpy during the day	0.724	0.525	0.731	0.534
Trouble getting out of bed in the morning	0.675	0.456	0.652	0.425
Fall back to sleep after being awakened	0.569	0.324	0.547	0.299
Need someone to awaken you	0.424	0.18		
How often do you think you need more sleep?	0.755	0.57	0.751	0.564
SS loadings	3.631		3.439	
Proportion Var	0.454		0.491	
Cronbach’s α	0.82		0.82	
McDonald’s ω	0.82		0.83	
	Average variance extracted — 37%		Average variance extracted — 41%	

*Note. F1 = unrotated standardized loadings on a factor. h2 is factor communality estimates. The regional, sex, and age effects on the PDSS total score.*

### The answers options were on a 5point Likert scale from 0 (never) to 4 (always)

The data in our study were collected across multiple data collection sites from different geographical regions in Russia. The descriptive statistics for different regions are presented in *Table 5.*

**Table 5:** Descriptive statistics for different regions of data collection

Region	Variable	n	mean	sd	median
Khabarovsk	Sex	559	1.56	0.5	2
	Age	559	13.55	1.47	13
	PDSS	559	14.35	5.7	14
Krasnoyarsk	Sex	326	1.6	0.49	2
	Age	326	15.07	1.33	15
	PDSS	326	12.78	5.04	13
Murmansk	Sex	145	1.5	0.5	1
	Age	145	14.63	1.31	14
	PDSS	145	13.23	6.12	13
Novosibirsk	Sex	424	1.53	0,5	2
	Age	424	14.4	1.42	14
	PDSS	424	12.64	5.59	12
Omsk	Sex	260	1.52	0.52	2
	Age	260	13.57	1.91	13
	PDSS	260	12.28	5.51	12
Perm’	Sex	86	1.58	0.5	2
	Age	86	15.21	1.59	15
	PDSS	86	11.6	5.54	11.5
Rostov-on-Don	Sex	767	1.52	0.5	2
	Age	767	14.98	1.41	15
	PDSS	767	1.52	0.5	2
Tatarstan	Sex	236	1.46	0.5	1
	Age	238	15.3	1.18	15
	PDSS	235	12.27	5.4	12
Tyumen’	Sex	549	1.55	0.53	2
	Age	549	14.23	2.01	14
	PDSS	549	11.97	5.68	12
Yaroslavl	Sex	229	1.57	0.5	2
	Age	229	15.44	1.23	16
	PDSS	229	13.31	5.54	13

We investigated whether the region of data collection affected the PDSS total score by applying multinomial logistic regression. The results of the multinomial logistic regression analysis are presented in *Table 6.* For multinomial logistic regression, the baseline variable should be set to compare different levels of nominal data. We used the Tyumen collection site as the baseline level. The relative ratio for a one-unit increase in the PDSS scores depending on the collection site ranged from 0.99 to 1.13 (a value of 1 means that there is no change, *Table 6*). The prediction accuracy of the regression model was 21.82%, indicating overall minor regional differences in PDSS scores.

**Table 6:** The results of multinomial logistic regression analysis

Region	(Intercept)	PDSS Score Corrected Odds Ratio	p-value
	0.000878	1.136154	0.308
Khabarovsk	0.360339	1.082254	0.1
Krasnoyarsk	0.385376	1.035529	0.19
Murmansk	0.175535	1.033744	0.093
Novosibirsk	0.537805	1.029662	0.34
Omsk	0.457738	1.002706	0.867
Perm’	0.2236	0.970294	0.232
Rostov-on-Don	1.002613	1.027197	0.25
Tatarstan	0.377662	1.010049	0.549
Yaroslavl	0.282636	1.032151	0.058

The next step of our analysis was to analyze the effects of age, sex, and time to took to fill out the questionnaire on the PDSS original and corrected scores. The zero-order Pearson’s correlations are presented in *Table 7.*

**Table 7:** Means, standard deviations, and correlations with confidence intervals

Variable	*M*	*SD*	1	2	3	4
1. Age	14.53	1.66				
2. Sex	46 % boys	0.50	0.02 [–0.01, 0.05]			
3. PDSS Score Corrected	12.80	5.68	0.11** [0.08, 0.14]	0.23** [0.19, 0.26]		
4. PDSS Score	14.14	6.32	0.08** [0,04, 0,11]	0.19** [0.16, 0.22]	0.98** [0.98, 0.98]	
5. Time of filling the questionnaire	13.64	3.93	–0.06** [–0.09, –0.02]	0.05** [0.02, 0.08]	0.01 [–0.02, 0.04]	0.01 –0.03, 0.04]

Note. M and SD are used to represent means and standard deviations, respectively. Values in square brackets indicate the 95% confidence interval for each correlation. * indicates p < 0.05. ** indicates p < 0.01.

**Figure 3.**
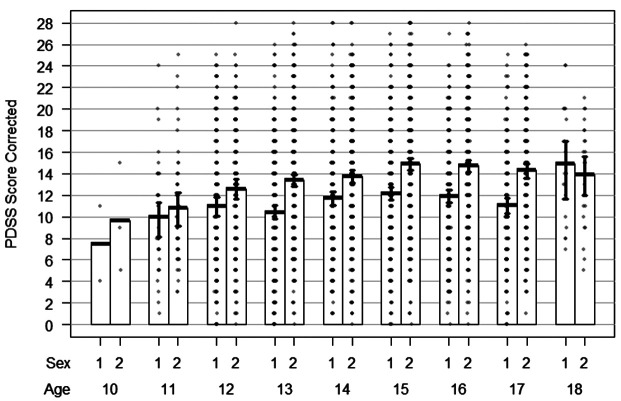
The age and sex differences in the PDSS corrected score

*Note. Bayesian Highest Density Intervals (HDIs) are presented as whiskers. For the “Sex” variable, 1 = boys, 2 = girls.*

The study results show that the age and sex of the participants were significantly correlated with the PDSS scores. The sex and age differences in the corrected PDSS scores are presented in *Figure 3.*

From *Figure 3*, it can be seen that 1) for age groups from 13 to 17 years, boys have lower PDSS scores than girls, and 2) there is a monotonic increase in PDSS scores from age 10 to 15, with a plateau afterward. Note that a higher PDSS score means more sleep-related problems.

## Discussion

In the current study, we investigated the psychometric properties of the Russian version of the PDSS questionnaire across a wide range of geographical regions in Russia. Our analysis revealed that the PDSS maintains the same unifactorial structure previously reported. We have also shown that although the PDSS was originally designed for participants of ages 1015, it demonstrates good psychometric quality for a broader age range, allowing it to be used for participants up to 18 years old.

Simultaneously, we found that improved psychometric characteristics of the questionnaire, compared to the adaptation of the PDSS by Randler and colleagues (2019), can be achieved by omitting one of the original items: “How often do you need someone to awaken you.” According to the IRT analysis, this item has limited value in differentiating participants in our study.

We hypothesize that this result may be associated with changes in the organization of morning routines since the PDSS questions were initially formulated. In contrast to the time when the original version of the questionnaire was developed (Drake et al., 2003), when it was common for parents or other adults to check on and wake up children if they were too sleepy, this responsibility is now largely delegated to alarm clocks on mobile phones that children typically possess. Excluding this item from the questionnaire increased the model fit and psychometric characteristics of the scale while preserving the same structure with only one factor. Overall, the construct validity of the instrument was demonstrated to be valid across various geographical regions and can be used for Russian-speaking youth samples to evaluate daytime sleepiness.

We observed no regional effects in the distribution of the PDSS total scores. In Russia, regional effects encompass seasonal variability in daylight. Previously, seasonal effects have been linked to disruptions in human behavior, most notably in mood and sleep patterns (Kasper et al. 1989). To date, only a few studies have investigated the potential seasonality of sleep and sleep problems, with the majority conducted in Nordic countries.

In one study, age-dependent effects of season on sleep (Thorleifsdottir et al. 2002) were identified for two specific items assessing 1) difficulties initiating and maintaining sleep, which were more frequent during the winter months, and 2) early morning awakenings, which were more common during the spring. However, one of the most recent and largest studies to date on the effects of extreme variation in natural illumination (the “Tromsø study,” Sivertsen et al., 2020) has demonstrated minimal influence of seasonality on sleep status. However, participants in that study were all over 40 years old, which limits its results’ generalizability to younger individuals. The lack of natural illumination effects on sleep quality in our study aligns with the previous findings of the Tromsø study (Sivertsen et al., 2020), indicating that these results apply not only to adults but also to schoolchildren.

In summary, our findings suggest that the extreme geographical properties of the data collection sites and potential seasonality effects were unlikely to impact the validity of the initial adaptation of the PDSS. It is possible that the more important factor is not the geographical characteristics of the participants’ location, but rather the individual differences in their circadian typology (Adan et al., 2006; Adan et al., 2012). However, this hypothesis requires further investigation.

We also analyzed the effects of participants’ age and sex on sleep quality. Our findings revealed both sex and age effects on sleep quality, with boys reporting fewer sleep-related problems than girls and younger children reporting fewer problems than older children. These age effects have been previously demonstrated in various studies (Alfano et al., 2009; Don et al., 2009; Owens et al., 2000) and have been attributed to factors ranging from developmental changes in the hormonal status and nervous system functioning in adolescents, to environmental effects such as shifts in academic responsibilities or the easing of daily regime requirements by parents. Adolescents also tend to go to bed later and wake much later than their adult or child counterparts due to changes in circadian rhythm and altered sleep drive, whereby the pressure to fall asleep accumulates more slowly in older adolescents (Galland et al., 2017).

The gender differences identified in our study are consistent with previous findings. Sleep disturbances have been reported to be more prevalent in girls after the onset of puberty and the beginning of the menstrual cycle (Johnson et al., 2006). However, it should be noted that Randler and colleagues (2019) did not report any gender differences, possibly due to their smaller sample size and the statistical power of their study.

## Conclusion

In conclusion, our study confirmed the validity and utility of the Russian version of the PDSS, enhancing the generalizability of this instrument. We have also replicated previously reported age and sex effects on sleep quality in schoolchildren. We recommend the widespread use of the PDSS for research and evaluation of daytime sleepiness and its influence, especially by health professionals, psychologists, and other specialists in Russia.
